# Ablation of the N-type calcium channel ameliorates diabetic nephropathy with improved glycemic control and reduced blood pressure

**DOI:** 10.1038/srep27192

**Published:** 2016-06-07

**Authors:** Shoko Ohno, Hideki Yokoi, Kiyoshi Mori, Masato Kasahara, Koichiro Kuwahara, Junji Fujikura, Masaki Naito, Takashige Kuwabara, Hirotaka Imamaki, Akira Ishii, Moin A. Saleem, Tomohiro Numata, Yasuo Mori, Kazuwa Nakao, Motoko Yanagita, Masashi Mukoyama

**Affiliations:** 1 Department of Nephrology, Kyoto University Graduate School of Medicine, Kyoto, Japan; 2 Medical Innovation Center, Kyoto University Graduate School of Medicine, Kyoto, Japan; 3 Institute for Clinical and Translational Science, Nara Medical University Hospital, Kashihara, Japan; 4 Department of Cardiovascular Medicine, Kyoto University Graduate School of Medicine, Kyoto, Japan; 5 Department of Diabetes, Endocrinology and Nutrition, Kyoto University Graduate School of Medicine, Kyoto, Japan; 6 Department of Nephrology, Kumamoto University Graduate School of Medical Sciences, Kumamoto, Japan; 7 Children’s Renal Unit, Bristol Royal Hospital for Children, University of Bristol, Bristol, UK; 8 Department of Synthetic Chemistry and Biological Chemistry, Kyoto University Graduate School of Engineering, Kyoto, Japan; 9 Department of Physiology, Fukuoka University Graduate School of Medical Sciences, Fukuoka, Japan

## Abstract

Pharmacological blockade of the N- and L-type calcium channel lessens renal injury in kidney disease patients. The significance of specific blockade of α1 subunit of N-type calcium channel, Ca_v_2.2, in diabetic nephropathy, however, remains to be clarified. To examine functional roles, we mated Ca_v_2.2^−/−^ mice with *db/db* (diabetic) mice on the C57BLKS background. Ca_v_2.2 was localized in glomeruli including podocytes and in distal tubular cells. Diabetic Ca_v_2.2^−/−^ mice significantly reduced urinary albumin excretion, glomerular hyperfiltration, blood glucose levels, histological deterioration and systolic blood pressure (SBP) with decreased urinary catecholamine compared to diabetic Ca_v_2.2^+/+^ mice. Interestingly, diabetic heterozygous Ca_v_2.2^+/−^ mice also decreased albuminuria, although they exhibited comparable systolic blood pressure, sympathetic nerve activity and creatinine clearance to diabetic Ca_v_2.2^+/+^ mice. Consistently, diabetic mice with cilnidipine, an N-/L-type calcium channel blocker, showed a reduction in albuminuria and improvement of glomerular changes compared to diabetic mice with nitrendipine. In cultured podocytes, depolarization-dependent calcium responses were decreased by ω-conotoxin, a Ca_v_2.2-specific inhibitor. Furthermore, reduction of nephrin by transforming growth factor-β (TGF-β) in podocytes was abolished with ω-conotoxin, cilnidipine or mitogen-activated protein kinase kinase inhibitor. In conclusion, Ca_v_2.2 inhibition exerts renoprotective effects against the progression of diabetic nephropathy, partly by protecting podocytes.

Diabetic nephropathy is the most common cause of end-stage renal failure^1^. To prevent the progression of diabetic nephropathy, a strict blood pressure control is strongly recommended^1^. Although renin-angiotensin system (RAS) inhibitors are extensively used as first-choice drugs for diabetic nephropathy^2^,^3^, the effects of other antihypertensive drugs on diabetic nephropathy remain elusive^4^.

Calcium channel blockers (CCBs) are frequently used in combination with RAS inhibitors because of their strong blood pressure-lowering properties and minimal adverse side effects^4^. The voltage-dependent calcium channels are localized in the plasma membrane and are essential for the release of neurotransmitters and hormones^5^. These channels are classified into L-, P/Q-, N-, R-, and T-type subtypes based on their pharmacological and electrophysiological properties. Molecular biological analysis has shown that calcium channels are composed of α1, α2/δ, β, and γ subunits^6^, among which α1 subunits are most important for defining channel properties. The α1 subunit genes have been cloned and classified into the following three subfamilies based on their sequence similarity: Ca_v_1.x, Ca_v_2.x, and Ca_v_3.x^7^. The α1b subunit Ca_v_2.2, encoded by the *CACNA1B* gene, is the only subunit which constitutes the N-type calcium channel.

Cilnidipine is an L-/N-type CCB which is used for patients with hypertension^8^. In several clinical^9^,^10^,^11^ and basic^12^,^13^,^14^ studies, cilnidipine has been shown to reduce proteinuria compared with other antihypertensive drugs. The CARTER study demonstrated that the L-/N-type CCB cilnidipine, but not the L-type CCB amlodipine, decreases urinary protein levels in RAS inhibitor-treated hypertensive patients with macroproteinuria^11^. The renoprotective effects of L-/N-type CCBs are at least partly due to the amelioration of glomerular hypertension^15^. L-type CCBs elicit afferent arteriole-prone vasodilation, which may increase the intraglomerular pressure^15^. On the other hand, L-/N-type CCBs ameliorate glomerular hypertension through the vasodilation of both afferent and efferent arterioles^16^.

The N-type calcium channel α1 subunit knockout (Ca_v_2.2^−/−^) mice, which lack the cytosolic portion of the N-type calcium channel, are viable and have an almost normal behavior but show a very low sympathetic nerve activity in atria^17^. Although previous reports used cilnidipine to inhibit N- and L-type calcium channel in diabetic nephropathy, molecular mechanisms of specific N-type calcium channel blockade in glomerular injury are not fully investigated. To address these questions, we investigated renal injury in N-type calcium channel-deficient *db/db* mice on the diabetes-prone C57BLKS/J background. In addition, we examined the functional role of N-type calcium channel in cultured podocytes.

## Results

### Characteristics of Diabetic Ca_v_2.2^−/−^ Mice

**Figure 1:**
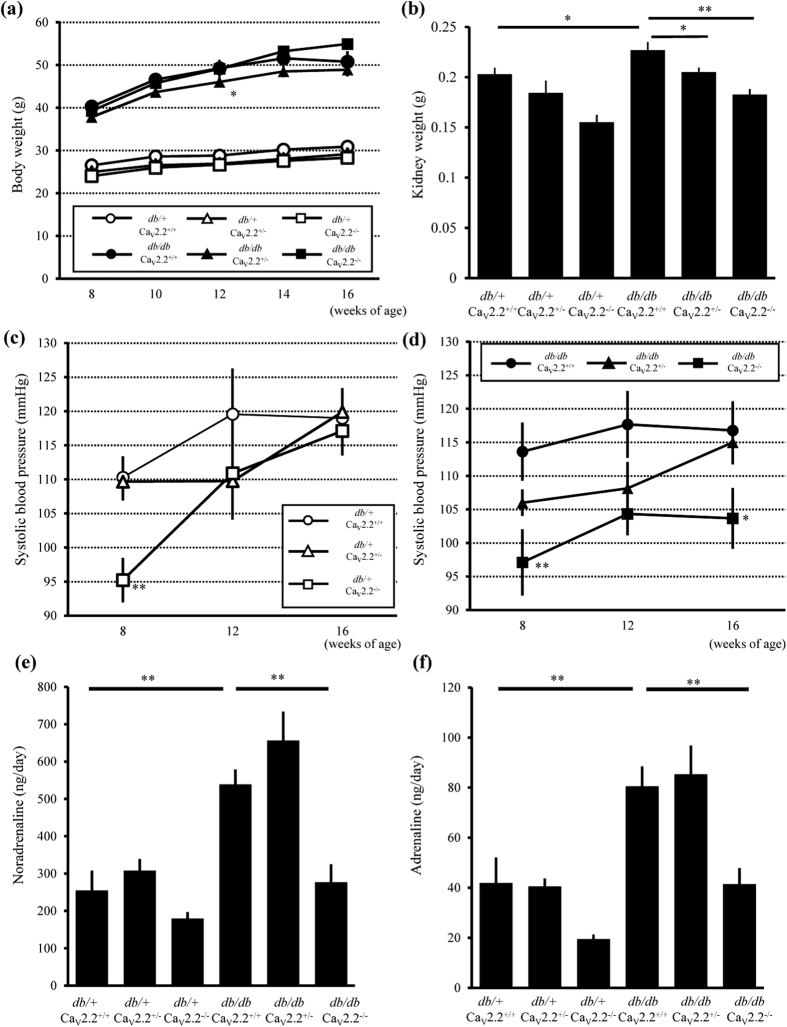
Body weight, kidney weights and systolic blood pressure. (**a**) Time course of body weight changes of experimental mice. Body weight in *db/db* mice significantly increased compared with *db*/+ mice. (**b**) Kidney weight of experimental mice at 16 weeks of age. *db/db* Ca_v_2.2^−/−^ mice showed less renal hypertrophy than that of *db/db* Ca_v_2.2^+/+^ mice. (**c**,**d**) Time course of systolic blood pressure changes of *db*/+ mouse groups (**c**) and *db/db* mouse groups (**d**). (**e**,**f**) Urinary catecholamine of experimental mice at 16 weeks of age. *db/db* Ca_v_2.2^−/−^ mice showed lower levels of urinary noradrenaline (**e**) and adrenaline (**f**) than *db/db* Ca_v_2.2^+/+^ mice. *db*/+ Ca_v_2.2^+/+^ mice (n = 7, white circles), *db*/+ Ca_v_2.2^+/−^ mice (n = 6, white triangles), *db*/+ Ca_v_2.2^−/−^ mice (n = 7, white squares), *db/db* Ca_v_2.2^+/+^ mice (n = 8, black circles), *db/db* Ca_v_2.2^+/−^ mice (n = 8, black triangles), and *db/db* Ca_v_2.2^−/−^ mice (n = 8, black squares). *p < 0.05, **p < 0.01 vs. Ca_v_2.2^+/+^ mice of the same *db* genotype.

To examine the role of the N-type calcium channel in diabetic nephropathy, we crossed Ca_v_2.2^−/−^ mice with *db*/*m* mice on the C57BLKS/J background more than six times to obtain *db/db* Ca_v_2.2^−/−^ mice. All diabetic (*db/db*) mouse groups showed significantly increased body weights compared with those of non-diabetic (*db*/+) controls, and *db/db* Ca_v_2.2^−/−^ mice tended to have a heavier body weight than other *db/db* mouse groups (Fig. 1a). *db/db* Ca_v_2.2^+/+^ mice showed renal hypertrophy, as indicated by an increase in kidney weight, whereas *db/db* Ca_v_2.2^−/−^ mice exhibited less renal hypertrophy (Fig. 1b). We measured the systolic blood pressure (SBP) of *db*/+ mice at 8, 12, and 16 weeks of age. *db*/+ Ca_v_2.2^−/−^ mice showed lower SBP than *db*/+ Ca_v_2.2^+/+^ mice at 8 weeks of age, and the difference disappeared at 12 and 16 weeks of age (Fig. 1c). On the other hand, the SBP of *db/db* Ca_v_2.2^−/−^ mice remained 15–20 mmHg lower than that of *db/db* Ca_v_2.2^+/+^ mice during the experimental period (Fig. 1d). These results indicate that the deficiency of Ca_v_2.2 resulted in reduction of basal SBP. To evaluate the mechanism of SBP reduction, we measured urinary catecholamine concentrations. *db/db* Ca_v_2.2^−/−^ mice, but not *db/db* Cav2.2^+/−^ mice, exhibited 50% lower levels of urinary noradrenaline and adrenaline than *db/db* Ca_v_2.2^+/+^ mice (Fig. 1e,f).

### Improvement of Glucose Metabolism in Ca_v_2.2^−/−^ Mice

**Figure 2:**
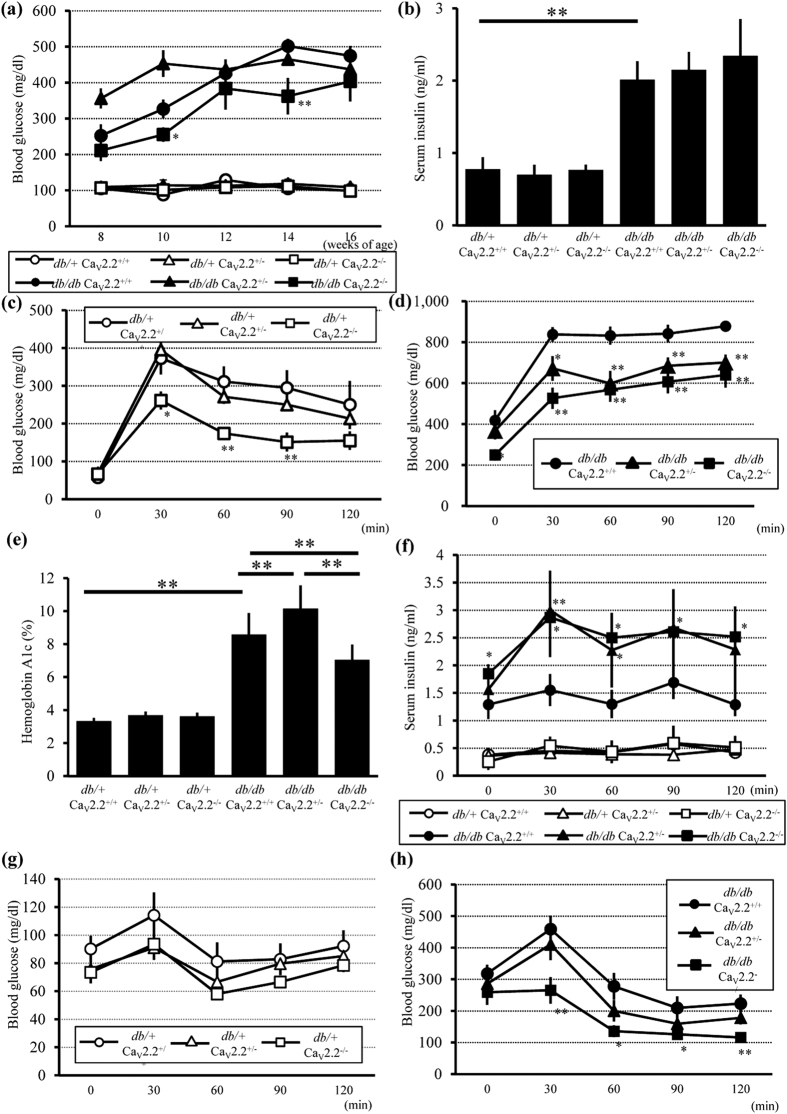
Blood glucose, serum insulin, and the results of GTTs and ITTs in *db*/+ and *db/db* mice. (**a**) Time course of 6-h fasting blood glucose concentrations. *db/db* Ca_v_2.2^−/−^ mice had lower levels of blood glucose than *db/db* Ca_v_2.2^+/+^ mice. (**b**) Serum insulin levels at 16 weeks of age. There were no different among *db/db* mice groups. (**c**) IPGTTs (2 g/kgBW) of *db*/+ mice at 15 weeks of age. *db*/+ Ca_v_2.2^−/−^ mice showed better glucose tolerance compared with *db*/+ Ca_v_2.2^+/+^ mice. (**d**) IPGTTs (1 g/kgBW) of *db/db* Ca_v_2.2^−/−^ mice at 15weeks of age. *db/db* Ca_v_2.2^−/−^ mice exhibited significantly reduced levels of blood glucose compared with *db/db* Ca_v_2.2^+/+^ mice. Since the glucometer has a detection limit up to 999 mg/dL, values above the detection limit were treated as 1000 mg/dL. (**e**) Hemoglobin A1c levels at 16 weeks of age. (**f**) Serum insulin levels of IPGTTs. The insulin levels in *db/db* Ca_v_2.2^−/−^ mice was significantly higher than these of *db/db* Ca_v_2.2^+/+^ mice. *db*/+ Ca_v_2.2^+/+^ mice (n = 6, white circles), *db*/+ Ca_v_2.2^+/−^ mice (n = 9, white triangles), *db*/+ Ca_v_2.2^−/−^ mice (n = 7, white squares), *db/db* Ca_v_2.2^+/+^ mice (n = 7, black circles), *db/db* Ca_v_2.2^+/−^ mice (n = 7, black triangles), and *db/db* Ca_v_2.2^−/−^ mice (n = 8, black squares) for GTT. (**g**,**h**) Blood glucose levels in ITTs at 15 weeks of age of *db*/+ (**g**) and *db/db* (**h**) mice. *db*/+ Ca_v_2.2^+/+^ mice (n = 5, white circles), *db*/+ Ca_v_2.2^+/−^ mice (n = 9, white triangles), *db*/+ Ca_v_2.2^−/−^ mice (n = 7, white squares), *db/db* Ca_v_2.2^+/+^ mice (n = 9, black circles), *db/db* Ca_v_2.2^+/−^ mice (n = 9, black triangles), and *db/db* Ca_v_2.2^−/−^ mice (n = 7, black squares) for ITT. *p < *0.05,* **p < *0.01* compared with Ca_v_2.2^+/+^ mice of the same *db* genotype.

To examine glucose metabolism in *db/db* Ca_v_2.2^−/−^ mice, we analyzed 6-h fasting blood glucose levels every 2 weeks during the experimental period. All *db/db* mouse groups showed hyperglycemia at 8 weeks of age, whereas all *db*/+ mouse groups had normal glucose levels (Fig. 2a). Notably, *db/db* Ca_v_2.2^−/−^ mice had or tended to have lower levels of blood glucose than *db/db* Ca_v_2.2^+/+^ mice during the experimental period (Fig. 2a). Sixteen-hour fasting serum insulin levels were not different among *db/db* mouse groups at 16 weeks of age (Fig. 2b). Intraperitoneal glucose tolerance tests (IPGTTs) were performed to further evaluate glucose metabolism at 15 weeks of age. The blood glucose levels in GTTs peaked at 30 min were 250 mg/dL in *db*/+ Ca_v_2.2^−/−^ mice and 400 mg/dL in *db*/+ Ca_v_2.2^+/+^ mice, indicating better glucose tolerance in *db*/+ Ca_v_2.2^−/−^ mice than that in *db*/+ Ca_v_2.2^+/+^ mice (Fig. 2c). *db/db* Ca_v_2.2^+/+^ mice developed severe glucose intolerance, whereas *db/db* Ca_v_2.2^−/−^ mice exhibited significantly reduced blood glucose levels compared with those in *db/db* Ca_v_2.2^+/+^ mice (Fig. 2d). The level of HbA1c was also reduced in *db/db* Ca_v_2.2^−/−^ mice compared with *db/db* Ca_v_2.2^+/+^ mice (Fig. 2e). Serum insulin levels of *db/db* mouse groups in GTTs were increased compared with those of *db*/+ mouse groups (Fig. 2f). There was no significant difference among *db*/+ mouse groups. On the other hand, the insulin levels in *db/db* Ca_v_2.2^−/−^ and *db/db* Ca_v_2.2^+/−^ mice were significantly higher than those of *db/db* Ca_v_2.2^+/+^ mice, suggesting that insulin secretion increased in mice with Ca_v_2.2 gene deletion.

Insulin tolerance tests (ITTs) were performed to determine whether the improved glucose tolerance observed in Ca_v_2.2^−/−^ mice was associated with increased insulin sensitivity. Ca_v_2.2^−/−^ mice showed lower glucose levels than Ca_v_2.2^+/+^ mice in both *db*/+ and *db/db* genotypes after insulin injection (Fig. 2g,h). These results indicate that deficiency of Ca_v_2.2 improves insulin secretion and insulin sensitivity in diabetic conditions.

### Reduced Urinary Albumin Excretion and Improved Hyperfiltration in Diabetic Ca_v_2.2^−/−^ Mice

**Figure 3:**
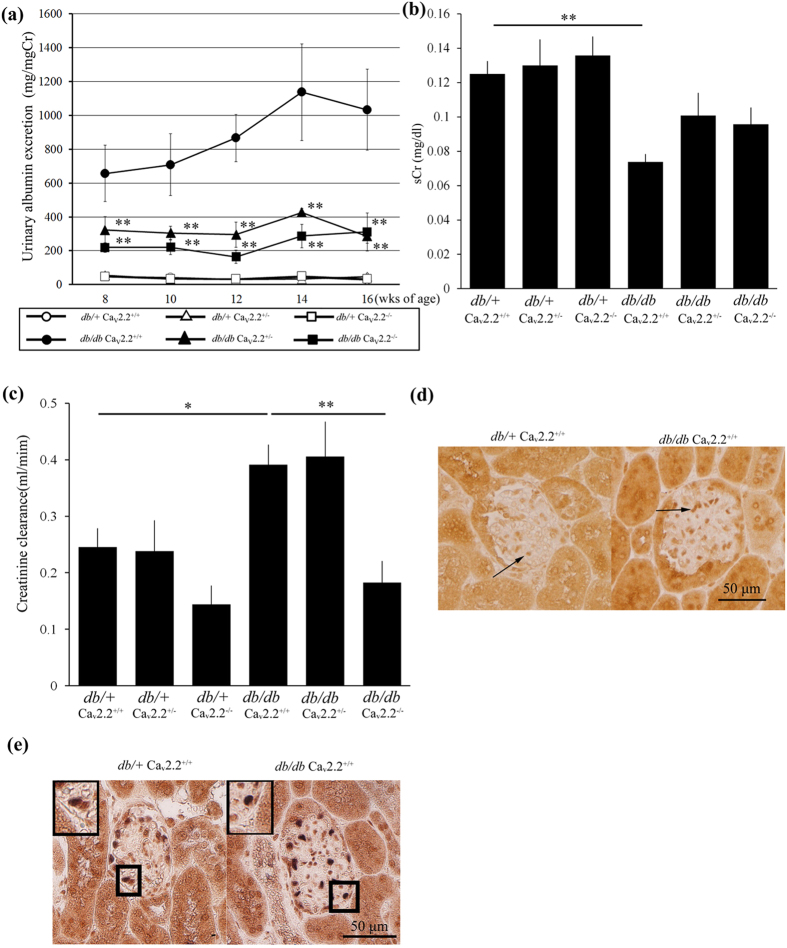
Role of Ca_v_2.2 on diabetic nephropathy. (**a**) Time course of urinary albumin excretion per milligram creatinine of experimental mice. Urinary albumin excretion of both *db/db* Ca_v_2.2^−/−^ mice and *db/db* Ca_v_2.2^+/−^ mice was lower than that of *db/db* Ca_v_2.2^+/+^ mice. (**b**) Serum creatinine levels at 16 weeks of age. (**c**) Creatinine clearance at 16 weeks of age. In *db/db* Ca_v_2.2^+/+^ mice, creatinine clearance was suppressed to the same level of *db/+* mice. (**d**) Immunohistochemical study of Ca_v_2.2 in *db*/+ Ca_v_2.2^+/+^ mice and *db/db* Ca_v_2.2^+/+^ mice. Ca_v_2.2 was positive at glomerular cells (arrows). (**e**) Double immunostaining for Ca_v_2.2 (brown) and WT1 (blue) shows double positive cells in a glomerulus (insets). *db*/+ Ca_v_2.2^+/−^ mice (white triangles), *db*/+ Ca_v_2.2^−/−^ mice (white squares), *db/db* Ca_v_2.2^+/+^ mice (black circles), *db/db* Ca_v_2.2^+/−^ mice (black triangles), and *db/db* Ca_v_2.2^−/−^ mice (black squares). *p < 0.05, **p < *0.01*, vs. *db/db* Ca_v_2.2^+/+^ mice. Scale bar = 50 μm.

To evaluate the functional alterations in the kidney of diabetic Ca_v_2.2^−/−^ mice, we examined urinary albumin excretion and serum creatinine level and calculated creatinine clearance (CCr). At baseline, there were no significant differences in urinary albumin excretion between *db*/+ Ca_v_2.2^+/+^ and *db*/+ Ca_v_2.2^−/−^ mice (Fig. 3a). Urinary albumin excretion markedly increased in *db/db* Ca_v_2.2^+/+^ at 8 weeks of age. In contrast, *db/db* Ca_v_2.2^−/−^ mice exhibited approximately 70% lower urinary albumin excretion than *db/db* Ca_v_2.2^+/+^ mice. Interestingly, *db/db* Ca_v_2.2^+/−^ mice also exhibited decreased albuminuria to the level comparable to that of *db/db* Ca_v_2.2^−/−^ mice (Fig. 3a). These results suggest that even a partial ablation of the N-type calcium channel leads to reduction in urinary albumin excretion.

Next, we examined the effect of N-type calcium channel ablation on hyperfiltration induced by diabetic milieu by measuring CCr. Basal levels of serum creatinine were not different regardless of the genotype of Ca_v_2.2 (Fig. 3b). *db/db* Ca_v_2.2^+/+^ mice showed a reduction of serum creatinine levels compared with those in *db*/+ Ca_v_2.2^+/+^ mice, thus indicating hyperfiltration in diabetic states (Fig. 3b). Although diabetic Ca_v_2.2^+/−^ mice exhibited CCr elevation because of hyperfiltration, the increase in CCr was almost completely abolished in *db/db* Ca_v_2.2^−/−^ mice, suggesting that hyperfiltration was normalized by deletion of the Ca_v_2.2 gene (Fig. 3c).

### N-type Calcium Channel Expression in Glomeruli of Control Mice and Renal Histological Improvement in Diabetic Ca_v_2.2^−/−^ Mice

N-type calcium channel localization was examined by an immunohistochemical study. Ca_v_2.2 was expressed in tubules and glomerular cells in the kidney of both *db*/+ Ca_v_2.2^+/+^ and *db/db* Ca_v_2.2^+/+^ mice (Fig. 3d). Double immunohistochemical staining showed that cells positive for Ca_v_2.2 in a glomerulus were also positive for WT1, a podocyte marker, indicating that Ca_v_2.2 is expressed in podocytes (Fig. 3e).

**Figure 4:**
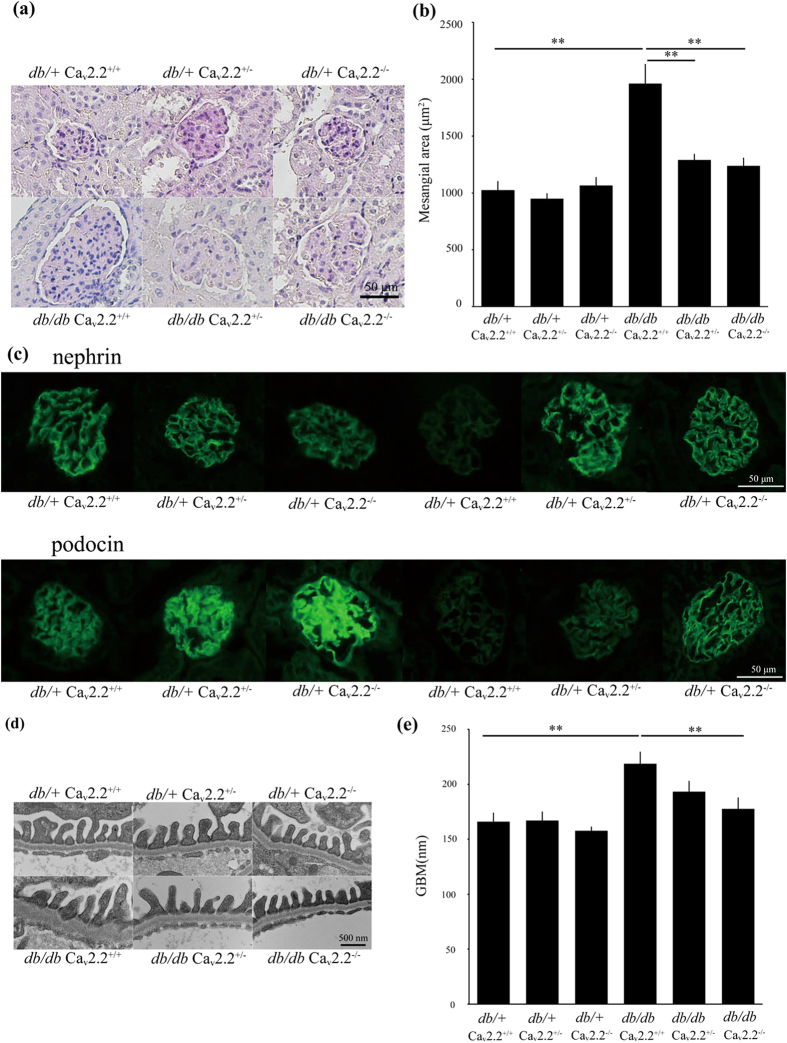
Histologic examination of a glomerulus. (**a**) Light microscopic analyses were performed at 16 weeks of age, stained with periodic acid-Schiff. *db/db* Ca_v_2.2^−/−^ mice showed reduced mesangial expansion compared with *db/db* Ca_v_2.2^+/+^ mice. Scale bar = 50 μm. (**b**) Mesangial area in a glomerulus at 16 weeks of age. Mesangial area was increased in *db/db* Ca_v_2.2^+/+^ mice and was suppressed in *db/db* Ca_v_2.2^−/−^ mice. (**c**) Immunostaining for nephrin and podocin. *db/db* Ca_v_2.2^−/−^ mice maintained of nephrin and podocin to the same level with *db*/+ mice. Scale bar = 50 μm. (**d**,**e**) Electron microscopic analyses of glomeruli of experimental mice at 16 weeks of age. GBM thickness was ameliorated in *db/db* Ca_v_2.2^−/−^ mice. Scale bar = 500 nm. **p < *0.01*, vs. *db/db* Ca_v_2.2^+/+^ mice.

We examined renal histology at 16 weeks of age. We observed mesangial expansion with glomerular hypertrophy in *db/db* Ca_v_2.2^+/+^ mice, which was consistent with diabetic alterations (Fig. 4a). In contrast, *db/db* Ca_v_2.2^−/−^ mice exhibited reduced glomerular mesangial expansion and inhibited glomerular hypertrophy compared with those seen in *db/db* Ca_v_2.2^+/+^ mice (Fig. 4a). *db/db* Ca_v_2.2^+/−^ mice also showed ameliorated glomerular changes. Morphometric analysis revealed that the mesangial area was increased in *db/db* Ca_v_2.2^+/+^ mice, whereas this increase was significantly suppressed in both *db/db* Ca_v_2.2^−/−^ and *db/db* Ca_v_2.2^+/−^ mice (Fig. 4b). These results suggest that N-type calcium channel ablation can limit the progression of diabetic nephropathy. Next, we evaluated podocyte injury in these mice. Immunostaining of nephrin and podocin, expressed predominantly in podocytes, was markedly decreased in *db/db* Ca_v_2.2^+/+^ mice compared with that in *db*/+ Ca_v_2.2^+/+^ mice (Fig. 4c). In contrast, *db/db* Ca_v_2.2^−/−^ mice maintained the expression of nephrin and podocin to the same level as *db*/+ mice, indicating the amelioration of podocyte injury (Fig. 4c). In electron microscopic analysis, *db/db* Ca_v_2.2^+/+^ mice showed thickening of the glomerular basement membrane (GBM) with slightly widened podocyte foot processes (Fig. 4d,e). GBM thickening was significantly ameliorated in *db/db* Ca_v_2.2^−/−^ mice (Fig. 4e).

### Glomerular Gene Expression and Phosphorylation of Extracellular Signal-Regulated Kinase (ERK) in Diabetic Ca_v_2.2^−/−^ Mice

**Figure 5:**
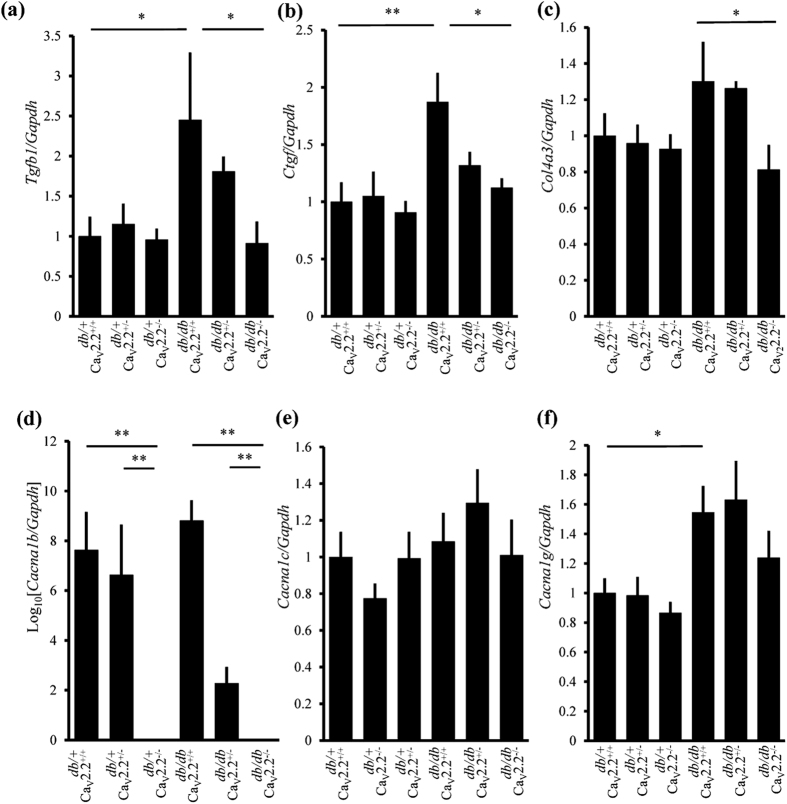
Analysis of glomerular mRNA expression. Real-time RT-PCR analyses of glomerular *Tgfb1* (**a**), *Ctgf* (**b**) *Col4a3* (**c**) *Cacna1b* (**d**) *Cacna1c* (**e**) and *Cacna1g* (**f**) were shown. *Gapdh* was used as control. *p < *0.05,* **p < *0.01*.

Analyses of the glomerular expression of extracellular matrix (ECM)-related genes revealed that TGF-β1 (*Tgfb1*) mRNA as well as connective tissue growth factor (*Ctgf*) mRNA were increased in *db/db* Ca_v_2.2^+/+^ mice, whose increase was significantly reduced in *db/db* Ca_v_2.2^−/−^ mice (Fig. 5a,b). Expression of pro-alpha 3 chain of collagen IV (*Col4a3*) mRNA was also significantly reduced in *db/db* Ca_v_2.2^−/−^ mice compared with *db/db* Ca_v_2.2^+/+^ mice (Fig. 5c). Gene expression of fibronectin (*Fn1*) and pro-alpha 1 chain of collagen I (*Col1a1*) tended to decrease in *db/db* Ca_v_2.2^−/−^ mice (Supplementary Fig. S1a,b). Glomerular gene expression of *Cacna1b*, Ca_v_2.2, was not altered in diabetic mice, was reduced in Ca_v_2.2^+/−^ mice and was not detected in Ca_v_2.2^−/−^ mice (Fig. 5d). Ca_v_2.2^+/−^ or Ca_v_2.2^−/−^ mice exhibited similar expression of *Cacna1c*, α1 subunit of L-type calcium channel as Ca_v_2.2^+/+^ mice (Fig. 5e). Glomerular expression of *Cana1g*, α1 subunit of T-type calcium channel was upregulated in diabetic mice, and was not different among diabetic or non-diabetic 3 groups (Fig. 5f).

**Figure 6:**
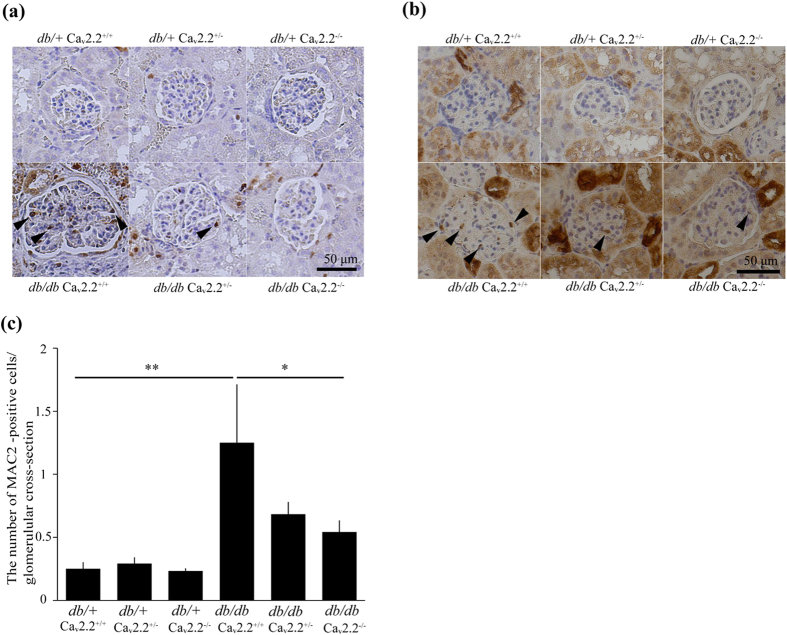
Immunohistochemical study for ERK and MAC2. (**a**) ERK phosphorylation was increased in a glomerulus, including mesangial cells and podocytes, of *db/db* Ca_v_2.2^+/+^ mice. Its increase was ameliorated in a glomerulus of *db/db* Ca_v_2.2^−/−^ mice. (**b**,**c**) Mac-2-positive cells in glomeruli increased in *db/db* Ca_v_2.2^+/+^ mice compared with those in *db*/+ Ca_v_ 2.2^+/+^ mice and suppressed in *db/db* Ca_v_2.2^−/−^ mice. **p < *0.01*. Scale bar = 50 μm.

Activation of extracellular signal-regulated kinase (ERK) has been shown to mediate TGF-β-induced accumulation of ECM protein in diabetic nephropathy^18^. We found that ERK phosphorylation was increased in glomeruli, including mesangial cells and podocytes, of *db/db* Ca_v_2.2^+/+^ mice compared with that of *db*/+ Ca_v_2.2^+/+^ mice (Fig. 6a). Phosphorylation of ERK was significantly lower in the glomeruli of *db/db* Ca_v_2.2^−/−^ mice than *db/db* Ca_v_2.2^+/+^ mice (Fig. 6a). Macrophages also play a critical role in the progression of diabetic nephropathy^19^. The immunohistochemical study showed that macrophage antigen-2 (Mac2)-positive cells in glomeruli increased by 3.0-fold in *db/db* Ca_v_2.2^+/+^ mice compared with those in *db*/+ Ca_v_ 2.2^+/+^ mice (Fig. 6b,c). This increase was significantly suppressed in *db/db* Ca_v_2.2^−/−^ mice (Fig. 6b,c).

### Pharmacological Inhibition of L- or N-type Calcium Channel Ameliorates Diabetic Nephropathy

To evaluate the pharmacological effect of N-type CCBs, we administered the N-/L-type CCB cilnidipine or the L-type CCB nitrendipine to *db/db* Ca_v_2.2^+/+^ mice and compared with *db/db* Ca_v_2.2^+/−^ and *db/db* Ca_v_2.2^−/−^ mice. There was no significant difference in body weight among vehicle-, nitrendipine-, cilnidipine-treated, *db/db* Ca_v_2.2^+/−^ and *db/db* Ca_v_2.2^−/−^ mouse groups (Supplementary Fig. S2a). Diabetic *db/db* Ca_v_2.2^−/−^ mice exhibited lower blood glucose level than *db/db* Ca_v_2.2^+/−^ mouse as shown previously (Fig. 2a, Supplementary Table S1). Diabetic mice with nitrendipine exhibited high urinary noradrenaline and adrenaline excretion, however, diabetic mice with cilnidipine did not change urinary catecholamine levels compared with those with nitrendipine (Supplementary Fig. S2b,c). Administration of nitrendipine or cilnidipine showed SBP almost similar to that in the vehicle and *db/db* Ca_v_2.2^+/−^ mouse groups (Fig. 1d, Supplementary Fig. S3a). Diabetic Ca_v_2.2^−/−^ mice showed lower SBP than vehicle, nitrendipine-, cilnidipine-treated and *db/db* Ca_v_2.2^+/−^ mice (Fig. 1d, Supplementary Fig. S3a). Urinary albumin excretion was suppressed in the cilnidipine-treated group and not in the nitrendipine-treated group, however, urinary albumin excretion in cilnidipine-treated mice was still higher than that in *db/db* Ca_v_2.2^+/−^ mice (Supplementary Fig. S3b). Renal histology showed that treatment with cilnidipine, but not with nitrendipine, inhibited mesangial expansion in diabetic mice to the comparable extent of *db/db* Ca_v_2.2^+/−^ mice (Supplementary Fig. S3c,d). In electron microscopic analysis, footprocesses widening and GBM thickening in diabetic mice was ameliorated only by cilnidipine treatment, which was comparable to *db/db* Ca_v_2.2^−/−^ mice (Supplementary Fig. S3e,f). Glomerular expression of TGF-β1 (*Tgfb1*) and connective tissue growth factor (*Ctgf*) mRNA tended to show a reduction in cilnidipine-treated mice, but the difference was not significant (Supplementary Fig. S4a,b). Accumulation of macrophages was decreased in cilnidipine-treated mice compared with vehicle-treated mice (Supplementary Fig. S4c,d).

### The Functional Role of N-type Calcium Channel on Cultured Podocytes

**Figure 7:**
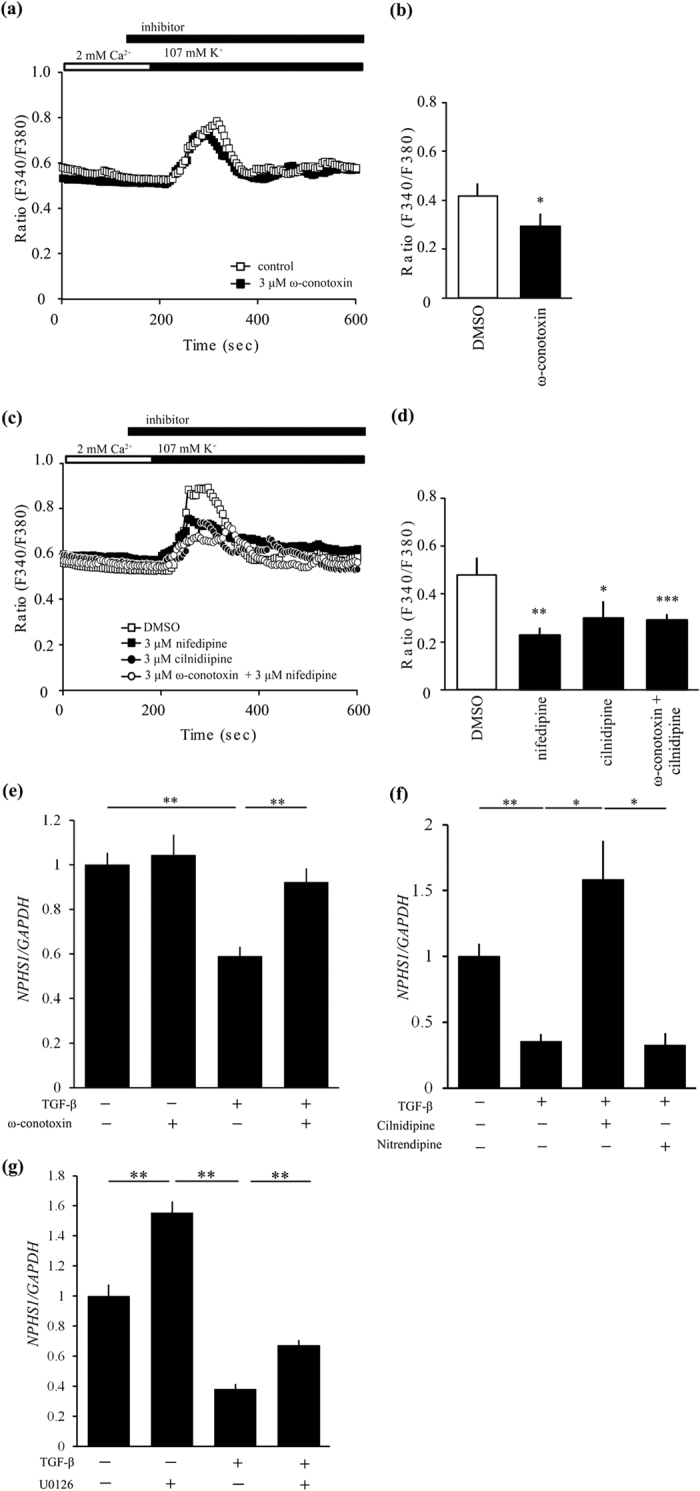
Expression of N-type calcium channels in cultured human podocytes and the functional role of these channels in depolarization-induced Ca^2+^ response. (**a**,**b**) Inhibitory effect of 3 μM ω-conotoxin (black squares) on depolarization-induced Ca^2+^ responses in human podocyte (n = 27–39) compared with control (white squares). (**a**) Averaged time courses of depolarization-induced Ca^2+^ response in human podocyte. (**b**) The bars represent the differences between the maximum of ration and the value of steady state. *p < 0.05 vs. control. (**c**,**d**) Inhibitory effect of 3 μM cilnidipine and 3 μM nifedipine on depolarization-induced Ca^2+^ responses in human podocyte (n = 20–30). (**c**) Averaged time courses of depolarization-induced Ca^2+^ response in human podocyte. 3 μM nifedipine; black squares, 3 μM cilnidipine; black circles, 3 μM ω-conotoxin + 3 μM nifedipine; white circles, DMSO; white squares. (**d**) The bars represent the differences between the maximum of ratio and the value of steady state. *p < 0.05, **p < 0.01, ***p < 0.001 vs. DMSO. (**e**) TGF-β1 (20 ng/ml) suppressed *NPHS1* expression in cultured human podocytes. Its inhibition was canceled by 100 nM ω-conotoxin. n = 11 for TGF-β (−) groups and n = 12 for TGF-β (+) groups. (**f**) Cilnidipine (10 μM) not nitrendipine (10 μM) ameliorated reduction of *NPHS1* expression induced by 5 ng/ml TGF-β1 in podocytes. Vehicle (n = 5) and other groups (n = 6, each). *p < 0.05, **p < 0.01. *GAPDH* was used as control.

First of all, to examine the role of the N-type calcium channel on podocytes, we measured intracellular Ca^2+^ ([Ca^2+^]_i_) concentration in cultured human podocytes. When podocytes were stimulated with 107 mM KCl, depolarization-dependent [Ca^2+^]_i_ increase was observed, and this [Ca^2+^]_i_ concentration was partially abolished by treatment with the N-type calcium channel blocker, ω-conotoxin (Fig. 7a,b). We also found that nifedipine, cilnidipine, or ω-conotoxin plus nifedipine inhibited depolarization-induced [Ca^2+^]_i_ in cultured podocytes (Fig. 7c,d). These results suggest that both N-type and L-type calcium channels are expressed in cultured human podocytes and are relevant to depolarization-induced [Ca^2+^]_i_ increase.

To confirm the effect of N-type calcium channel blockade on podocytes, we examined the changes in nephrin expression in cultured human podocytes treated with ω-conotoxin. Administration of exogenous TGF-β resulted in a decreased expression of nephrin in podocytes (Fig. 7e). This decrease was significantly reversed by pre-incubation with ω-conotoxin (Fig. 7e). Nitrendipine did not change the TGF-β-induced reduction of nephrin expression, but cilnidipine upregulated nephrin expression in podocytes (Fig. 7f).

Finally, we revealed that inhibition of ERK by mitogen-activated kinase kinase (MEK) inhibitor U0126 significantly ameliorated TGF-β-induce reduction of nephrin expression (Fig. 7g).

## Discussion

Investigation of diabetic nephropathy in rodents is rendered difficult partly by the lack of adequate animal models displaying typical diabetic nephropathy^20^. Among these limited mouse models of diabetic nephropathy, *db/db* mice are one of the most frequently used disease models. Nevertheless, the genetic background plays an important role in developing diabetic nephropathy; e.g., *db/db* mice on the C57BLKS background exhibit massive proteinuria and mesangial expansion, whereas *db/db* mice on the C57BL/6J show less severe glomerular and glycemic changes^20^,^21^. Knockout mice are mostly generated on the C57BL/6J or 129/SvJ backgrounds^22^. In order to overcome these situations, we backcrossed Ca_v_2.2 knockout mice on the C57BL/6J background with C57BLKS to explore the role of the N-type calcium channel in diabetic nephropathy.

The present study demonstrated that glycemic control was improved with enhanced insulin secretion in diabetic mice by ablation of the N-type calcium channel. In a previous study, Ca_v_2.2^−/−^ mice showed lower fasting glucose levels and better glucose tolerance than wild-type mice without any change in insulin sensitivity upon GTTs^23^. The same study reported that, after 10 weeks of high-fat diet feeding, Ca_v_2.2^−/−^ mice still showed lower fasting glucose levels and better glucose tolerance than Ca_v_2.2^+/+^ and Ca_v_2.2^+/−^ mice. The mechanisms how Ca_v_2.2 deletion resulted in better glycemic control have not been clarified yet, but another report has shown that the N-type calcium channel is present on pancreatic α cells and that GLP-1 inhibits glucagon release by selectively suppressing this channel^24^. In our study, *db/db* Ca_v_2.2^+/−^ mice showed a marginally improved glucose tolerance; however, the reduction in urinary albumin excretion was much larger than expected from the degree of glycemic control, suggesting that mechanisms other than the amelioration in glucose metabolism would contribute to renoprotective effects, particularly in *db/db* Ca_v_2.2^+/−^ mice.

Diabetic Ca_v_2.2^−/−^ mice showed lower SBP with a marked reduction in urinary catecholamine levels. Similarly, a previous report showed that Ca_v_2.2^−/−^ mice exhibited lower SBP than control mice because of vasodilatation, reduction of heart contractile activity, and inhibition of sympathetic nerve activity^25^. Most notably in our study, urinary albumin excretion was reduced by 70% and renal hyperfiltration was normalized in *db/db* Ca_v_2.2^−/−^ mice. In addition, even diabetic Ca_v_2.2^+/−^ mice, in which the expression of the N-type calcium channel is ~50% less than wild-type mice, showed a decrease in albuminuria to the level comparable to that in diabetic Ca_v_2.2^−/−^ mice. The former denied reduction in sympathetic nerve activity, suggesting that partial inhibition of the N-type calcium channel could ameliorate albuminuria without affecting sympathetic nerve function.

Cilnidipine has been shown to ameliorate glomerular hypertrophy by dilating both afferent and efferent arterioles in the kidney^12^,^16^, and L-type calcium channel blockade does not improve glomerular hypertension because the L-type calcium channel blockade mainly dilates afferent arterioles^15^. Because N-type calcium channels exist at synaptic nerve endings^26^ in both the afferent and efferent arterioles, and the blockade of the N-type calcium channel inhibits norepinephrine release^26^, such sympatholytic effect of the N-type calcium channel ablation may have worked to ameliorate glomerular injury in our study. Cilnidipine showed similar antihypertensive effects and suppression of proteinuria both in innervated and denervated spontaneous hypertensive rat (SHR)^27^, thus suggesting that renal sympathetic nerves may have a limited contribution to its renoprotective effects. In our study, albuminuria as well as glomerular histological changes were significantly alleviated in *db/db* Ca_v_2.2^+/−^ mice and cilnidipine-treated diabetic mice without reduction of urinary catecholamine levels, further providing a possibility for mechanisms independent of the sympathetic nerve activity. In addition, we showed no compensatory expression of α1 subunits of L- and T- type calcium channel in Ca_v_2.2 heterozygous or knockout mice. Further study is necessary to distinguish the effects of N- and T- type calcium channel blocker, because blockade of T-/L-type calcium channel by manidipine^28^ or efonidipine^29^ exhibit similar effects as N-/L-type calcium channel blocker, cilnidipine, in terms of amelioration of glomerular hypertension.

As diabetic N-type calcium channel knockout mice improved metabolic parameters including glycemic control and blood pressure, the amelioration of diabetic renal injury in N-type calcium channel knockout mice is partly due to simultaneous improvements of metabolic parameters. However, in addition to these actions, we showed possible functional roles of the N-type calcium channel in podocytes. Previous reports showed that glomerular podocytes express the N-type calcium channel^13^,^27^. Fan *et al*. demonstrated the immunoreactivity of N-type calcium channels in kidney vascular walls, possibly in the nerves in adventitia, distal tubules, and podocytes, and that the N-type calcium channel in cultured podocytes was involved in the angiotensin II-induced production of reactive oxygen species^13^. In the present study, we also found that the N-type calcium channel is expressed in glomeruli, presumably podocytes, in control mice. The genetic inhibition of N-type calcium channels reduced podocyte injury in diabetic mice. *In vitro* analysis using calcium imaging revealed that N-type calcium channels as well as L-type calcium channels are functional in depolarization-induced [Ca^2+^]_i_ increase in cultured human podocytes. Furthermore, we examined the changes in nephrin expression induced by TGF-β stimulation in cultured human podocytes and revealed that the decrease caused by the administration of exogenous TGF-β was canceled by pre-incubation with ω-conotoxin, cilnidipine or MEK inhibitor. High glucose-induced ERK activation in podocytes is closely associated with diabetic nephropathy through the protein kinase C pathway^30^,^31^, suggesting that ERK plays an important role in TGF-β-induced podocyte injury.

In conclusion, we have demonstrated that the ablation or blockade of the N-type calcium channel in diabetic mice exerts renoprotective effects, which effects may be brought about by both improvement of metabolic parameters and protection from podocyte injury. These results indicate that the N-type calcium channel works as an aggravating factor in a mouse model of diabetic nephropathy, suggesting a possibility that the N-type calcium channel should provide a promising therapeutic target for preventing the progression of diabetic nephropathy in humans.

## Methods

### Animals and drug treatment

All animal experiments were approved by the Animal Experimentation Committee of Kyoto University Graduate School of Medicine and were carried out in accordance with the approved guidelines. Mice deficient in the α_1B_ subunit of the N-type calcium channel (Ca_v_2.2^−/−^ mice) were produced on the 129/SvJ background^17^ and then backcrossed with C57BL/6J mice more than 10 times. BKS.Dg-*Dock*7^m^ +/+ *Lepr*^db^/J mice (*db/m*) mice on the C57BLKS/J background were purchased from Clea Japan Co. Ltd (Tokyo, Japan). Ca_v_2.2^−/−^ mice were backcrossed with *db*/*m* mice on C57BLKS/J more than six times. We prepared six groups as follows: *db*/+ Ca_v_2.2^+/+^ mice, *db*/+ Ca_v_2.2^+/−^ mice, *db*/+ Ca_v_2.2^−/−^ mice, *db/db* Ca_v_2.2^+/+^ mice, *db/db* Ca_v_2.2^+/−^ mice, and *db/db* Ca_v_2.2^−/−^ mice. Male diabetic *db/db* mice and their non-diabetic *db*/+ mice (control) were used in this study. Blood pressure was measured by the tail-cuff method (MK-2000ST; Muromachi Kikai, Tokyo, Japan) every 4 weeks^32^. Urine samples were collected using metabolic cages every 2 weeks for measurement of creatinine and albumin^28^. Thereafter, mice were sacrificed at 16 weeks of age.

Nitrendipine (15 mg/kg/day; a gift from Tanabe Mitsubishi pharmaceutical company), cilnidipine (15 mg/kg/day; a gift from Mochida pharmaceutical company) or vehicle were mixed with powdered food at 150–187.5 μg/g, the concentration of which is adjusted by weekly food intake. CCBs were administered to *db/db* Ca_v_2.2^+/+^ mice from 9 weeks to 16 weeks of age.

### Blood and urine parameter measurements

Serum and urinary creatinine levels were assayed by an enzymatic method (SRL, Tokyo, Japan)^32^. Blood glucose was measured with Gunze Life Check (Gunze, Tokyo, Japan)^33^. Plasma insulin levels were measured by enzyme immunoassay (Ultra-sensitive PLUS mouse insulin kit, Morinaga, Yokohama, Japan)^34^. For GTTs, after 16-h fasting, *db*/+ mice and *db/db* mice received *ip* injections with 2 or 1 g/kg glucose, respectively^34^. For insulin tolerance tests (ITTs), after 16-h fasting, *db*/+ mice and *db/db* mice received *ip* injections with 0.4 and 2 units/kg human insulin (Novo Nordisk, Bagsvaerd, Denmark), respectively^34^. We measured catecholamines in 24-h urine by adding 1 mL 6N HCl per 100 ml of urine. Urinary catecholamine contents were measured by high-performance liquid chromatography (SRL, Tokyo, Japan). Urinary albumin was measured with murine albumin enzyme-linked immunosorbent assay (ELISA) kit (Exocell, Philadelphia, PA)^32^. HbA1c values were determined as described previously^33^.

### Renal histology, immunohistochemistry, and electron microscopy

Histologic and immunohistochemical examinations were performed as described previously^32^. Immunofluorescence analyses for nephrin and podocin were performed as described previously^32^. Immunohistochemical studies for phospho-ERK and Mac2 (also known as lectin galactoside-binding soluble 3, LGALS3) were performed as described^19^,^32^. For the double immunohistochemical study for Wilms’ tumor factor-1 (WT1) and Ca_v_2.2, kidney sections were fixed with Brasil’s fixative. Sections were boiled for 10 min for antigen retrieval. After blocking, sections were incubated with both goat anti-WT-1 antibody (Santa Cruz Biotechnology, Santa Cruz, CA) and rabbit anti-Ca_v_2.2 antibody (Alomone Labs, Jerusalem, Israel) for 1 h at room temperature. Then, sections were incubated with alkaline phosphatase-conjugated anti-goat IgG and peroxidase-conjugated anti-rabbit IgG (Jackson ImmunoResearch, West Grove, PA) for 1 h. Electron microscopy analysis was performed as described previously^32^. The GBM thickness was measured with the Image J software (Ver 1.45; National Institutes of Health, Bethesda, MD).

### Real-time RT-PCR analysis

Total RNA was extracted using AllPrep DNA/RNA/Protein Mini Kit (Qiagen, Germantown, MD) from glomeruli that were isolated by the graded sieving method^32^. Quantitative real-time PCR was performed using Premix Ex Taq (Takara Bio, Otsu, Japan) on the StepOnePlus system (Applied Biosystems, Foster City, CA) as described previously^35^. Glomerular expression of *Tgfb1, Ctgf, Col4a3, Cacna1b, Cacna1c, Cacna1g, Fn1 and Col1a1* mRNA was evaluated. Some of primers and probe sets were described elsewhere^32^ and as follows: *Cacna1b* forward primer, 5′-gagactccaggggctgacac-3′; *Cacna1b* reverse primer, 5′-cgttcagtggcctcctccg-3′; *Cacna1b* probe, 5′-FAM-cagtagacgtcaccaccggcgcg-TAMRA-3′; *Cacna1c* forward primer, 5′-agactgagtctgtcaacactga-3′; *Cacna1c* reverse primer, 5′-ggagatccgatgggcaagc-3′; *Cacna1c* probe, 5′-FAM-ttctccctcgatgtcacctccagcca-TAMRA-3′; *Cacna1g* forward primer, 5′-ccgagagatccctaggacaca-3′; *Cacna1g* reverse primer, 5′-gtgtctgctggttgggagtg-3′; *Cacna1g* probe, 5′-FAM-cccaaagcccagtcaggctccatctt-TAMRA-3′. Expression of each mRNA was normalized with GAPDH mRNA (TaqMan rodent GAPDH control reagents; Applied Biosystems).

### Cell culture

Conditionally immortalized human podocytes were cultured as described previously^36^,^37^. Differentiated podocytes were serum-starved for 24 h and then pretreated with 100 nM ω-conotoxin (Peptide Institute, Osaka, Japan)^38^, 10 μM cilnidipine or 10 μM nitrendipine at 1 h before the stimulation of recombinant human TGF-β1 (R&D Systems, Minneapolis, MN). Cells were harvested after 24 h, and the expression level of nephrin was assayed by real-time RT-PCR analysis (n = 5–12).

### Calcium imaging

Calcium imaging was performed as described previously with some modification^39^. Human podocyte cells were plated onto poly-L-lysine-coated glass coverslips and subjected to measurement 3–16 h after plating on the coverslips. The cells on coverslips were loaded with fura-2 in RPMI 1640 containing 1 μM fura-2-acetoxymethyl ester (fura-2-AM; Dojindo Laboratories, Kumamoto, Japan), 10% fetal bovine serum (Sigma), 10 μg/mL insulin, 5.5 μg/mL transferrin, 5 ng/mL selenium (Sigma), 30 units/mL penicillin, and 30 μg/mL streptomycin at 37 °C for 30 min. The coverslips were then plated in a perfusion chamber mounted on the stage of the microscope. The fura-2 fluorescence images of the cells were recorded in HEPES-buffered saline (HBS, 2 mM Ca^2+^) (in mM): 107 NaCl, 6 KCl, 1.2 MgSO_4_, 2 CaCl_2_, 1.2 KH_2_PO_4_, 11.5 glucose, and 20 HEPES (pH 7.4 adjusted with NaOH). Four minutes after image acquisition, the cells were stimulated with 107 mM K^+^ solution (in mM): 107 KCl, 6 NaCl, 1.2 MgSO_4_, 2 CaCl_2_, 1.2 KH_2_PO_4_, 11.5 glucose, and 20 HEPES (pH 7.4 adjusted with NaOH). All the reagents dissolved in water or dimethylsulfoxide were diluted to their final concentrations and applied to the cells by perfusion. Fluorescence images of the cells were recorded and analyzed with a video image analysis system (AQUACOSMOS; Hamamatsu Photonics, Shizuoka, Japan). The ratio of the fluorescence intensity at 340 nm to the intensity at 380 nm was calculated to evaluate the change in intracellular calcium levels.

### Statistical analysis

Data are expressed as the mean ± SEM. Statistical analysis was performed using one-way ANOVA. *P* < 0.05 was considered statistically significant.

## Additional Information

**How to cite this article**: Ohno, S. *et al*. Ablation of the N-type calcium channel ameliorates diabetic nephropathy with improved glycemic control and reduced blood pressure. *Sci. Rep.* **6**, 27192; doi: 10.1038/srep27192 (2016).

## Supplementary Material

Supplementary Information
