# Supplementary material for: Ablation of the N-type calcium channel ameliorates diabetic nephropathy with improved glycemic control and reduced blood pressure
Source: Sci Rep. 2016 Jun 7;6:27192. doi: 10.1038/srep27192 (PMC4895143; doi:10.1038/srep27192)
Supplement: Supplementary Information [file srep27192-s1.pdf]

Ablation of the N-type calcium channel ameliorates diabetic nephropathy with improved glycemic control and reduced blood pressure

Shoko Ohno[1], Hideki Yokoi[1*], Kiyoshi Mori[2], Masato Kasahara[3], Koichiro Kuwahara[4], Junji Fujikura[5], Masaki Naito[5], Takashige Kuwabara[1,6], Hirotaka Imamaki[1], Akira Ishii[1], Moin A. Saleem[7], Tomohiro Numata[8,9], Yasuo Mori[8], Kazuwa Nakao[2], Motoko Yanagita[1], Masashi Mukoyama[1,6]

[1]Department of Nephrology, [2]Medical Innovation Center, [4]Department of Cardiovascular Medicine, [5]Department of Diabetes, Endocrinology and Nutrition, Kyoto University Graduate School of Medicine, Kyoto, Japan, [3]Institute for Clinical and Translational Science, Nara Medical University Hospital, Kashihara, Japan, [6]Department of Nephrology, Kumamoto University Graduate School of Medical Sciences, Kumamoto, Japan, [7]Academic Renal Unit, Bristol Children's Hospital, University of Bristol, Bristol, UK, [8]Department of Synthetic Chemistry and Biological Chemistry, Kyoto University Graduate School of Engineering, Kyoto, Japan, and [9]Department of Physiology, Fukuoka University Graduate School of Medical Sciences, Fukuoka, Japan

***Corresponding author:** Hideki Yokoi, M.D., Ph.D., Department of Nephrology, Kyoto University Graduate School of Medicine, 54 Shogoin Kawahara-cho, Sakyo-ku, Kyoto 606-8507, Japan.

Phone: +81-75-751-4286, **Fax:** +81-75-771-9452, **e-mail:** yokoih@kuhp.kyoto-u.ac.jp

Supplementary Table S1. Blood glucose level (mg/dl)

Weeks of age	vehicle	nitrendipine	cilnidipine	*db/db* $Ca_v2.2^{+/-}$	*db/db* $Ca_v2.2^{-/-}$
8w	252.25 ± 31.67	254.6 ± 26.16	276.57 ± 34.96	356.13 ± 27.81†,‡	211 ± 29.29¶¶
10w	326.63 ± 26.16	339.78 ± 34.49	396.14 ± 52.97	453 ± 37.2†,	255.38 ± 20.08¶¶,‡‡,§§
12w	426.13 ± 36.36	350.33 ± 39.43	432.57 ± 65.42	436.5 ± 28.29	383.75 ± 59.14
14w	501.88 ± 20.24	401.4 ± 28.25	508.14 ± 87.75	465.13 ± 18.43	362.38 ± 50.75†,§
16w	474.75 ± 27.47	453.67 ± 15.68	533.29 ± 66.68	437 ± 25.25	403.33 ± 55.88§

†$P < 0.05$, ††$P < 0.01$, vs. vehicle-treated mice

‡$P < 0.05$, ‡‡$P < 0.01$, vs. nitrendipine-treated mice

§$P < 0.05$, §§$P < 0.01$ vs. cilnidipine-treated mice

¶¶$P < 0.01$, vs. *db/db* $Ca_v2.2^{+/-}$

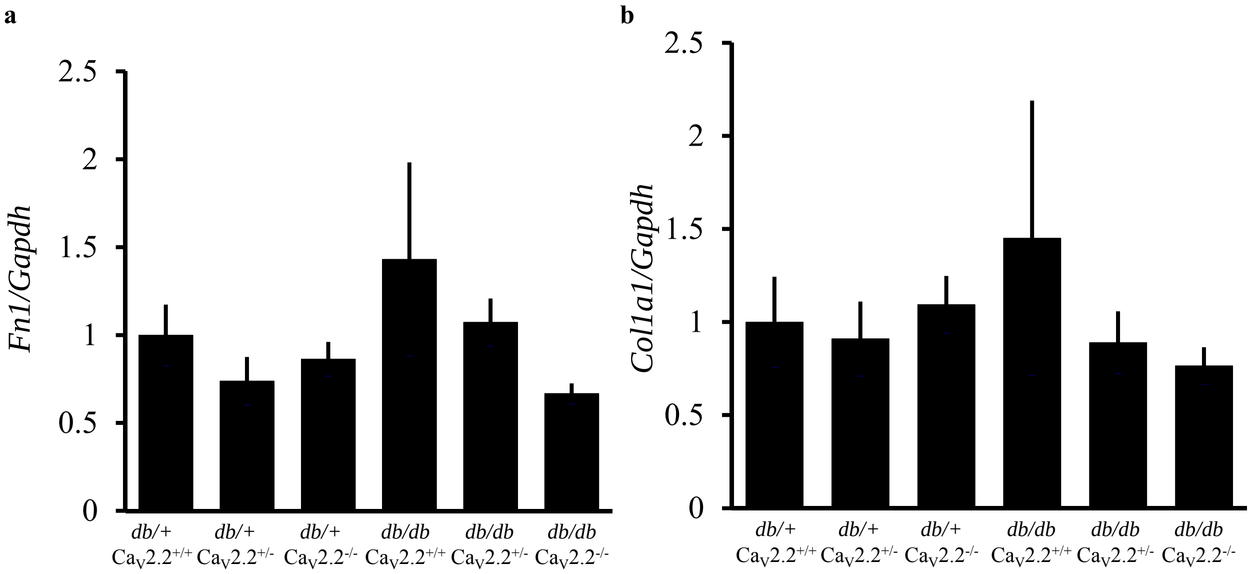

Supplementary Figure S1. Glomerular mRNA expression. Expression of *Fn1* (a) and *Col1a1* (b). *Gapdh* was used as internal control . n = 8, each.

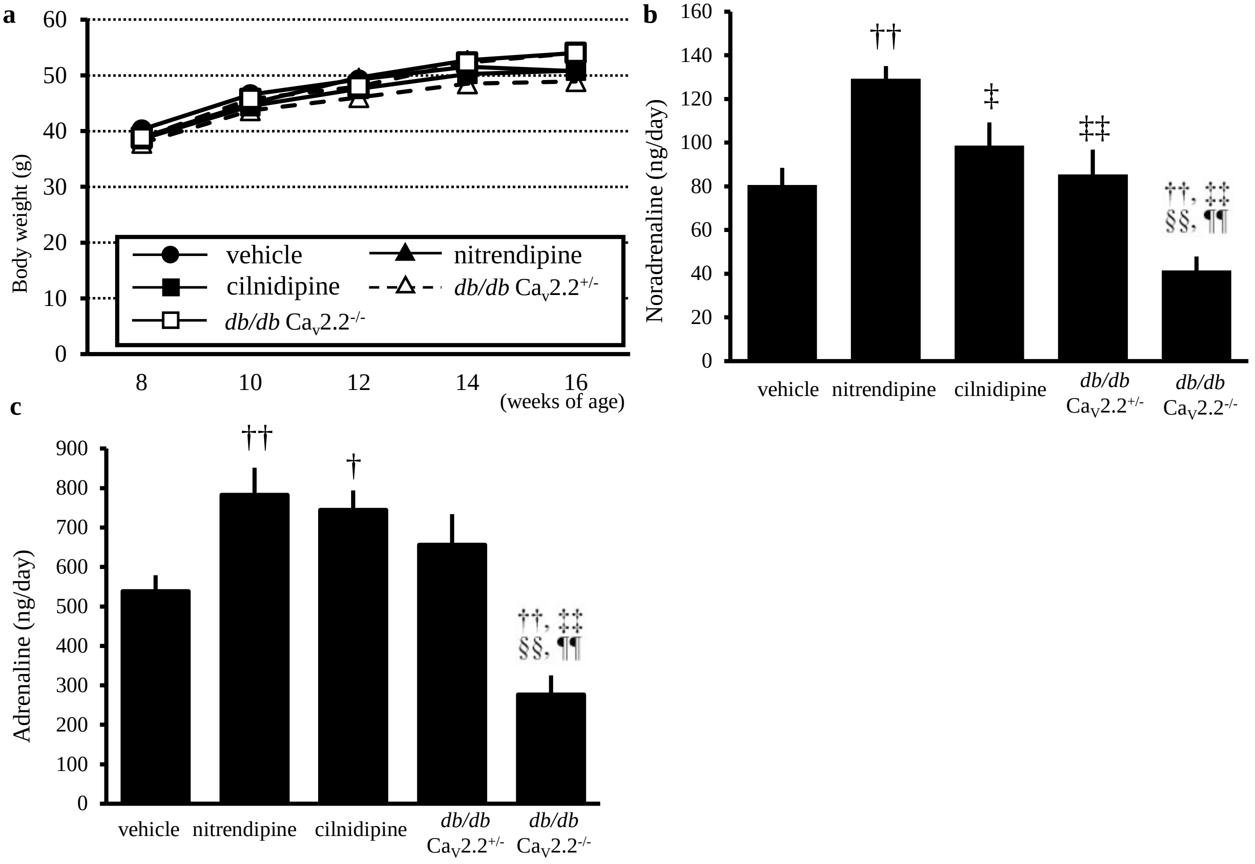

Supplementary Figure S2. **Metabolic parameters among vehicle-, nitrendipine-, cilnidipine-treated groups.** Body weight (a) in the experiments. No significant difference between groups. Urinary noradrenaline (b) and adrenaline (c) of experimental mice at 16 weeks of age. Vehicle, n = 8; nitrendipine; n = 9, cilnidipine, n = 7. Data of *db/db* Ca$_v$2.2$^{+/-}$ mice (n = 8) and *db/db* Ca$_v$2.2$^{-/-}$ mice (n = 8) were shown for comparison. †$P < 0.05$, ††$P < 0.01$ vs. vehicle-treated mice, ‡$P < 0.05$, ‡‡$P < 0.01$ vs. nitrendipine-treated mice, §§ $P < 0.01$ vs. cilnidipine-treated mice, ¶¶$P < 0.01$ vs. *db/db* Ca$_v$2.2$^{+/-}$ mice.

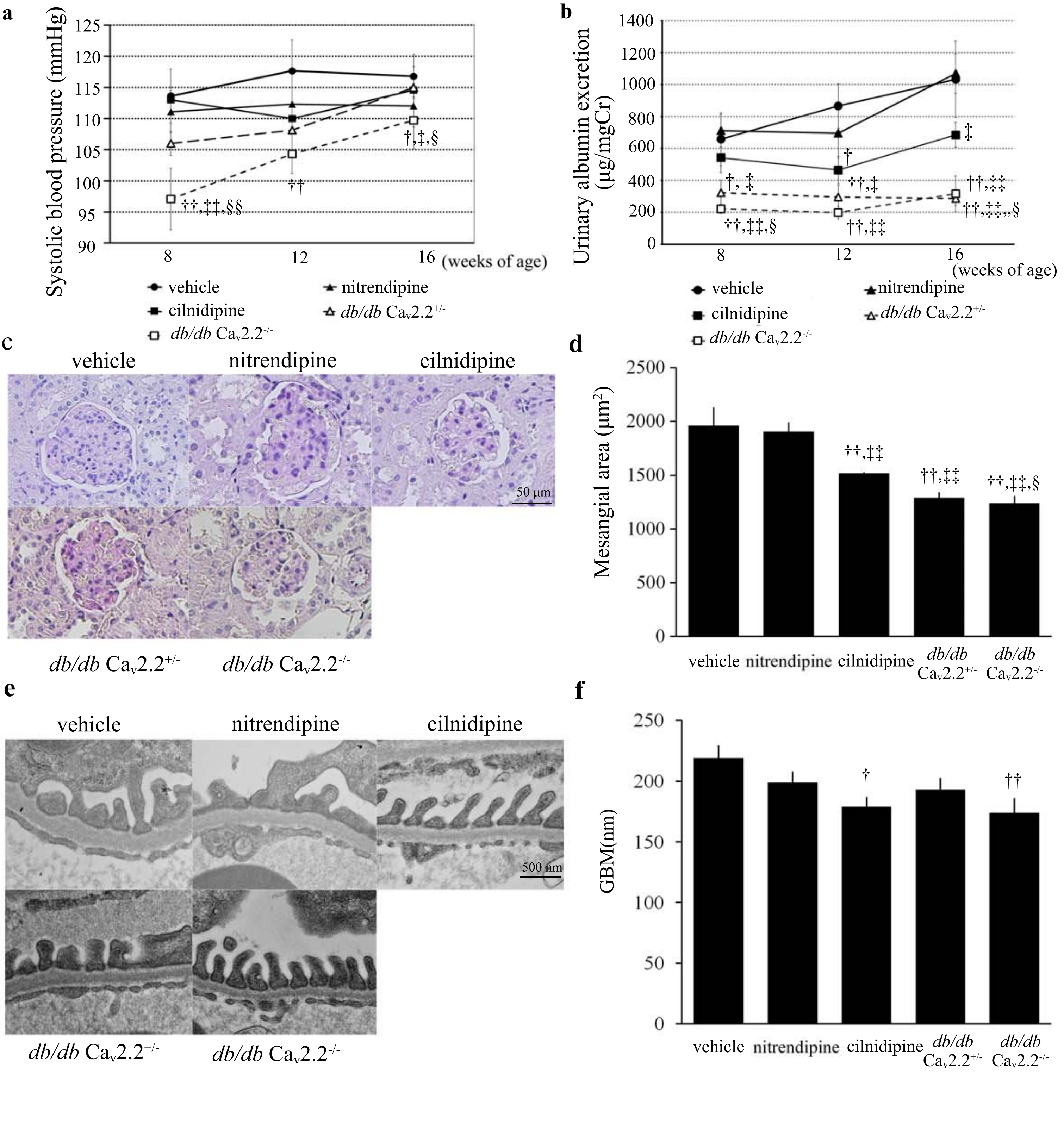

Supplementary Figure S3. Systolic blood pressure, urinary albumin excretion and histologic examination of glomeruli among vehicle-, nitrendipine- and cilnidipine-treated groups. (a) Time course of systolic blood pressure change. Administration of nitrendipine or cilnidipine reduced SBP to the same level. (b) Time course of urinary albumin excretion. (c and d) Light microscopic analyses were performed at 16weeks of age, stained with periodic acid-Schiff. (e and f) Electron microscopic analyses of glomeruli of experimental mice at 16 weeks of age. Vehicle (n = 8, black circles), nitrendipine (n = 9, black triangles), cilnidipine (n = 7, black squares). The data of *db/db* $Ca_v2.2^{-/-}$ mice (n = 8) and *db/db* $Ca_v2.2^{+/-}$ mice (n = 8) were shown for the comparison.

†$P < 0.05$, ††$P < 0.01$ vs. vehicle-treated mice; ‡$P < 0.05$, ‡‡$P < 0.01$ vs. nitrendipine-treated mice; §$P < 0.05$ vs. cilnidipine-treated mice.

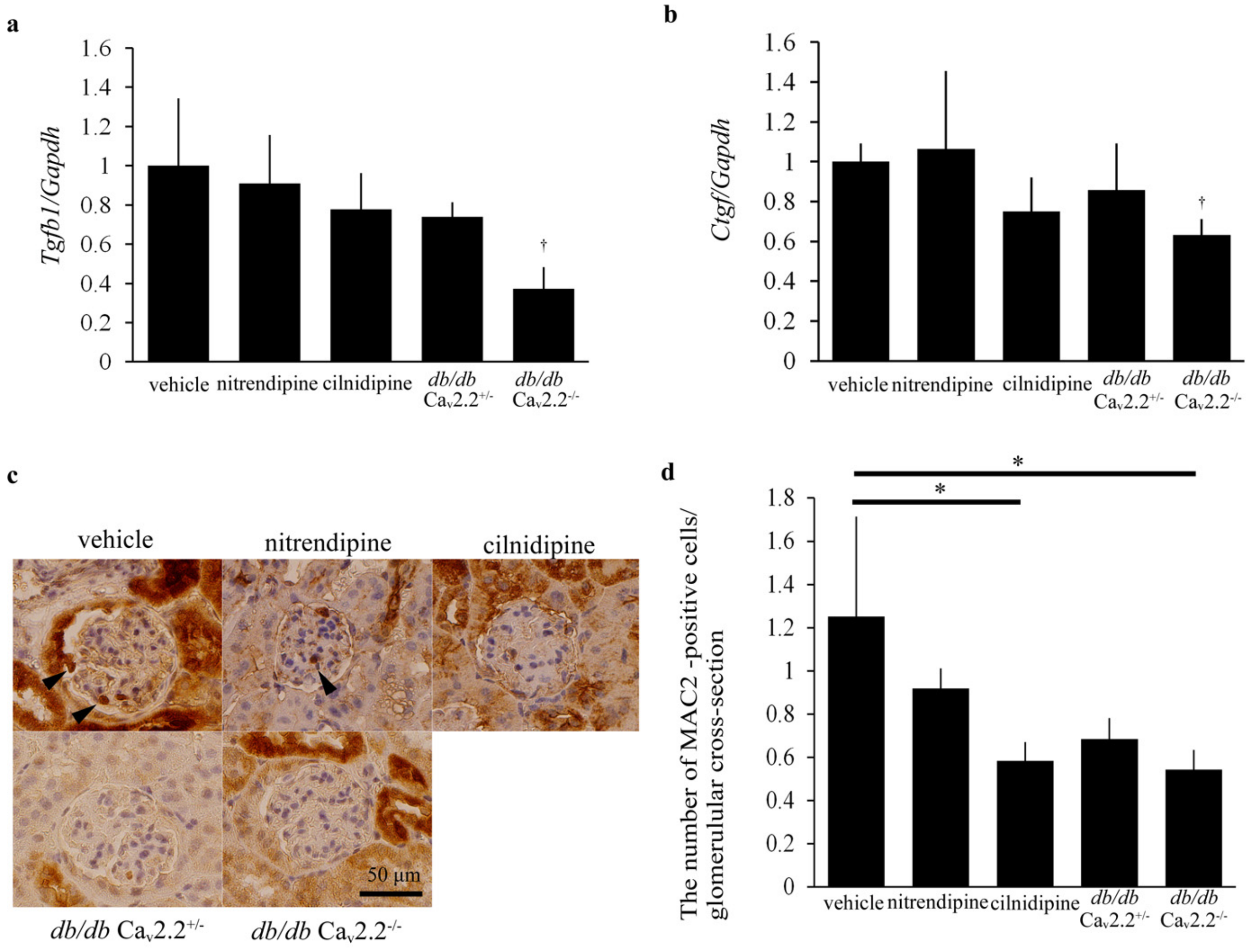

Supplementary Figure S4. Glomerular mRNA expression and immunohistochemical study for MAC2.
Real-time RT-PCR analysis of *Tgfb1* (a) and *Ctgf* (b) in glomeruli. Gapdh was usd as control.
(c) Immunohistochemicalstudy for MAC2. (d) The number of MAC2-positive cells per glomeular section .
†$P < 0.05$ vs.vehicle-treated mice,*$P < 0.05$.
